# Use of Transcriptomic Analyses to Elucidate the Mechanism Governing Nodal Root Development in *Eremochloa ophiuroides* (Munro) Hack.

**DOI:** 10.3389/fpls.2021.659830

**Published:** 2021-04-23

**Authors:** Rui Wang, Haoyan Zhao, Hailin Guo, Junqin Zong, Jianjian Li, Haoran Wang, Jianxiu Liu, Jingjing Wang

**Affiliations:** The National Forestry and Grassland Administration Engineering Research Center for Germplasm Innovation and Utilization of Warm-season Turfgrasses, Institute of Botany, Jiangsu Province and Chinese Academy of Sciences, Nanjing, China

**Keywords:** centipedegrass, nodal root, plant hormone, E3 ubiquitin-protein ligase, SINAT5

## Abstract

Centipedegrass [*Eremochloa ophiuroides* (Munro) Hack.] is a perennial warm-season grass that originated in China, and its speed of nodal rooting is important for lawn establishment. In our study, centipedegrass nodal rooting ability was limited by node aging. Transcriptome sequencing of nodal roots after 0, 2, 4, and 8 days of water culture was performed to investigate the molecular mechanisms of root development. GO enrichment and KEGG pathway analyses of DEGs indicated that plant hormone signal transduction and transcription factors might play important roles in centipedegrass nodal root growth. Among them, E3 ubiquitin-protein ligases participated in multiple hormone signal transduction pathways and interacted with transcription factors. Furthermore, an E3 ubiquitin protein ligase *EoSINAT5* overexpressed in rice resulted in longer roots and more numerous root tips, while knockout of *LOC_Os07g46560* (the homologous gene of *EoSINAT5* in rice) resulted in shorter roots and fewer root tips. These results indicated that *EoSINAT5* and its homologous gene are able to promote nodal root development. This research presents the transcriptomic analyses of centipedegrass nodal roots, and may contribute to elucidating the mechanism governing the development of nodal roots and facilitates the use of molecular breeding in improving rooting ability.

## Introduction

Adventitious roots develop post-embryonically from non-root tissues in the majority of monocotyledon fibrous root systems (Atkinson et al., 2014). Adventitious roots include junction roots, nodal roots, prop/stem roots, and stress-induced roots (Steffens and Rasmussen, 2016). Adventitious roots can be naturally induced as an adaptation to environmental changes, such as flooding (Visser et al., 1996; Lorbiecke and Sauter, 1999) and dark–light transitions (Sorin et al., 2005; Gutierrez et al., 2009), and they can also be induced artificially by cutting and/or hormone application (Ahkami et al., 2009; Sukumar et al., 2013). Some economically important crops (such as strawberries and sweet potatoes) and most warm-season turfgrasses [such as *Cynodon dactylon* (L.) *Pers.*, *Zoysia japonica Steud*, *Eremochloa ophiuroides* (Munro) Hack., and *Paspalum vaginatum* Sw.] have specialized stolons and adventitious roots produced by stem nodes, which are known as nodal adventitious roots; as a result, these plants can propagate rapidly.

Adventitious rooting in different species has different regulatory mechanisms, which in turn exhibit different physiological mechanisms and functions (Hochholdinger et al., 2004; Atkinson et al., 2014; Bellini et al., 2014; Pacurar et al., 2014). At present, the research works of nodal roots are mostly focused on maize, sorghum, and other food crops with erect stems. For example, fewer crown nodal roots of maize can improve nitrogen uptake and nitrate efficient (Guo and York, 2019), and more aerial nodal roots may improve root-lodging resistance efficiently (Zhang et al., 2018). Comparing with seminal roots, nodal roots have larger metaxylem area and higher levels of auxin, which may enhance the uptake of nutrients transported with the flow of water (Liu et al., 2020). In sorghum, the growth angle of nodal roots strongly influences the spatial distribution of soil profile to impact drought adaptation (Joshi et al., 2017). For nodal roots of stolon, the research works were mostly focused on white clover indicating that nodal roots influence branch development (Thomas et al., 2003). However, the molecular mechanism of stolon nodal roots largely remains unknown.

Centipedegrass [*E. ophiuroides* (Munro) Hack.] is a perennial warm-season grass that originated in China and has the characteristics of good adaptation to poor soil, low maintenance, few pests, and high ornamental value (Li et al., 2020). Centipedegrass has well-developed stolons, and the rooting ability of stolon nodal roots is related to the speed of lawn establishment. However, the regulatory mechanism governing centipedegrass nodal root development has rarely been reported. Previous studies confirmed that phytohormones are the most important modulators of root development and auxin plays a central role (Lavenus et al., 2013). Auxin has effects on every aspect of root development, including meristem initiation, emergence, and elongation (Bellini et al., 2014). For example, auxin transporter PIN-FORMED (PIN) proteins are important in the regulation of root development in rice and maize (Xu et al., 2005; Li Z. et al., 2018). In rice, plants transgenically expressing RNA that interfere with the *OsPIN1* gene have the same number of adventitious root primordia as the wild type but rarely germinate adventitious roots, indicating that the *PIN1* gene is involved in the germination of adventitious roots (Xu et al., 2005). *ZmPIN1a* overexpression in maize results in the formation of longer seminal roots and denser lateral roots with an increasing number of lateral roots and also inhibits root elongation (Li Z. et al., 2018).

Ubiquitin-mediated proteolysis is involved in auxin signal transduction to influence root development (Frugis and Chua, 2002). E3 ubiquitin-protein ligases determine the specific recognition of target proteins, interact with transcription factors, and degrade them (Xie et al., 2002; Kelley and Estelle, 2012). In rice, the RING finger E3 ubiquitin ligase soil-surface rooting 1 (SOR1) protein interacts with the Aux/IAA proteins OsIAA9 and OsIAA26 (Chen et al., 2018). OsIAA26 is the target protein of OsSOR1 and can be degraded by the ubiquitin/26S proteasome (Chen et al., 2018). However, OsIAA9 inhibits the E3 activity of OsSOR1 to protect OsIAA26 from degradation, indicating that the OsSOR1–OsIAA26 module functions downstream of OsTIR1/AFB2-auxin-OsIAA9 signaling to regulate ethylene inhibition of rice seed root growth (Chen et al., 2018). In apple, a small ubiquitin-like modifier (SUMO)-conjugating E2 ligase MdSCE1 interacts with MdARF8, and the conjugating enzyme activity of MdSCE1 is enhanced by the E3 ligase MdSIZ1 to form an MdSCE1–MdSIZ1–MdARF8 complex, which regulates lateral root formation (Zhang et al., 2020). Lateral root formation is promoted by overexpressing *MdSIZ1* or *MdARF8* in transgenic apple plants (Zhang et al., 2020). The *Arabidopsis* RING finger-containing E3 ligase SINAT5 attenuates the auxin signal by interacting with and degrading NAC1 (NAM/CUC-like protein 1) to reduce the number of lateral roots (Xie et al., 2000, 2002).

Centipedegrass is an excellent warm-season turfgrass (Li J. et al., 2018), and the stolon is the main reproductive organ of warm-season turfgrass. However, the weak rooting ability of centipedegrass stolon nodal roots influences the speed of lawn establishment, and the molecular mechanism governing centipedegrass nodal root development has not been fully elucidated. In this study, we evaluated the rooting ability of different nodes of centipedegrass accession E039, and sampled the first node roots at four time points (0, 2, 4, and 8 days) for transcriptome sequencing. The differentially expressed genes (DEGs) were identified by Gene Ontology (GO) and Kyoto Encyclopedia of Genes and Genomes (KEGG) analyses. One E3 ubiquitin-protein ligase *EoSINAT5* was selected from DEGs and its effects on nodal root development were verified in transgenic rice. This study provided an overview of centipedegrass nodal root development and helped to elucidate the molecular mechanisms governing this process.

## Materials and Methods

### Plant Materials and Treatments

The centipedegrass accession E039, “Ganbei,” which was collected from Mount Lushan of Jiangxi Province in China (28°36′N, 116°00′E), was stored in a greenhouse at the Institute of Botany, Jiangsu Province and the Chinese Academy of Sciences. For the rooting ability evaluation of different order nodes, the rooting rates of 1–16 nodes (from the young nodes to the old nodes) of E039 were counted after 4, 8, and 12 days of water culture with pure water. Each sample consisted of 15 biological replications. The rooting rate of nodes was calculated according to the following equation:

$$rootingrateofnodes=\frac{\text{root}\text{number}}{\text{node}\text{number}}\times 100\%$$

The rooting rate of branches was calculated according to the following equation:

$$rootingrateofbranchecs=\frac{\text{root}\text{number}}{\text{branch}\text{number}}\times 100\%$$

### RNA-Seq and Bioinformatic Analysis

The roots of the first node of E039 were sampled at 0 day (the root length about 0.1 cm), 2 days (the root length about 0.5 cm), 4 days (the root length about 2.0 cm), and 8 days (the root length about 4.0 cm) after water culture, frozen by liquid nitrogen, and stored at −80°C. Each sample had three biological replicates. Total RNA was extracted using an RNA Isolation Kit (Waryong, Beijing, China). A total of 12 cDNA libraries were constructed, and the transcriptome was sequenced by Novogene (Tianjin, China)^1^ on an Illumina HiSeq 2500 platform. The raw datasets are available in the NCBI repository http://www.ncbi.nlm.nih.gov/bioproject/PRJNA687624. Clean reads were obtained from the raw data by removing adaptor sequences, reads with ambiguous bases “N,” low-quality reads (padj < 10) and fragments measuring fewer than 20 bp in length. The transcriptome was assembled based on the left.fq and right.fq using Trinity (v2.4.0) software (Grabherr et al., 2011). Gene function was annotated based on Nr (NCBI non-redundant protein sequences), Nt (NCBI non-redundant nucleotide sequences), Pfam (Protein family), KOG/COG (Clusters of Orthologous Groups of proteins), Swiss-Prot (a manually annotated and reviewed protein sequence database), KO (KEGG Ortholog database), and GO. The DEGs were identified from each comparison through the DESeq (Wang et al., 2020) R package with padj < 0.05 and | log2FoldChange| > 1. GO enrichment analysis of DEGs was performed by the GOseq R package (Young et al., 2010). The DEGs were identified from the KEGG^2^ enrichment analyses, and KOBAS (Mao et al., 2005) software was used to test the statistical enrichment of DEGs. All heat maps were created using TBtools software (Chen et al., 2020) with the “Log Scale” and “Row Scale.” The trend analysis of DEGs was performed by the online OmicShare tool^3^.

### Quantitative RT-PCR Validation

Twelve DEGs were randomly selected from Supplementary Table 1 to validate the reliability of the transcriptome data. The primers for these genes were designed using Primer 5.0 software. The *EoActin* was used as a housekeeping gene (Chung et al., 2019). Each sample was analyzed with three biological and three technical replicates, and the relative expression levels were calculated using the 2^–ΔΔCT^ method (Livak and Schmittgen, 2001). The primers used in this study are listed in Supplementary Table 2.

### EoSINAT5 Genetic Transformation

On the basis of the *Cluster-13984.84258* sequence in the centipedegrass transcriptome, the EoSINAT5-F/R primer pair was designed with Primer 5.0 software, and the integrated coding sequence (CDS) and DNA sequences were obtained (Supplementary Table 2). The sequence of *AtSINAT5* was obtained from the TAIR website (*AT5G53360*)^4^. Gene structure analysis of *EoSINAT5* and *AtSINAT5* was performed using GSDS 2.0^5^. The amino acid sequences of other SINAT5 proteins in other species were obtained from the NCBI website^6^. The protein alignment of EoSINAT5 and other SINAT5s was performed with DNAMAN 5.2.2 software.

The CDS of *EoSINAT5* was first cloned into a pMD19-T vector and subsequently introduced into the pEarleyGate103 vector by LR recombination. The pEarleyGate103 vector has the herbicide Basta resistance gene *Bar*. The gRNA target sequence (GGGGCAGCGGTTGTGAACCC) of *LOC_Os07g46560* (the homologous gene of *EoSINAT5* in rice) was prepared in oligodimers and subsequently introduced into the pYLCRISPR/Cas9-MH vector. The pEarleyGate103-*EoSINAT5* and pYLCRISPR/Cas9-MH-*LOC_Os07g46560* were introduced into “Nipponbare” rice by BIOGLE GeneTech (Hangzhou Biogle Co., Ltd., Zhejiang, China). The detected primers of overexpression transgenic lines were *Bar*-F/R, while the knocked out transgenic lines were *LOC_Os07g46560*-F/R (Supplementary Table 2). *OsActin* (GenBank: XM_015774830.2) was used as a housekeeping gene. The total root length and tip number of wild-type and transgenic lines (each line contained ten plants) were analyzed by WinRHIZO (Zealquest Scientific Technology Co., Ltd.). The significant difference analysis was performed by SPSS Statistics v.18.0 (Duncan’s test) (SPSS Inc., Chicago, IL, United States).

## Results

### Rooting Ability Evaluation of Different Nodes

**FIGURE 1:**
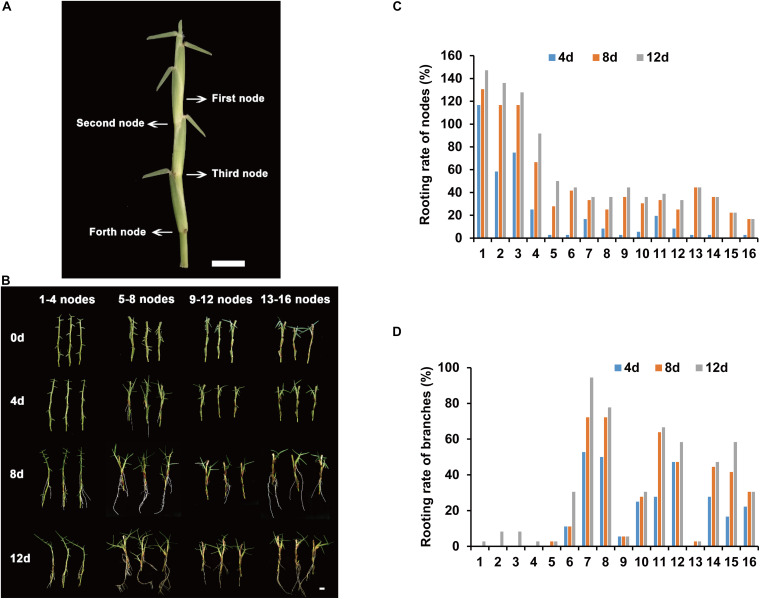
Rooting ability evaluation of different accessions and nodes. **(A)** The nodes order of centipedegrass. *Scale bar* represents 1 cm. **(B)** The rooting situation of nodes 1–16 of E039 after water culture at 0, 4, 8, and 12 days. **(C)** The rooting rate of 1–16 nodes after water culture at 4, 8, and 12 days. **(D)** The rooting rate of branches of nodes 1–16 after water culture at 4, 8, and 12 days.

**FIGURE 2:**
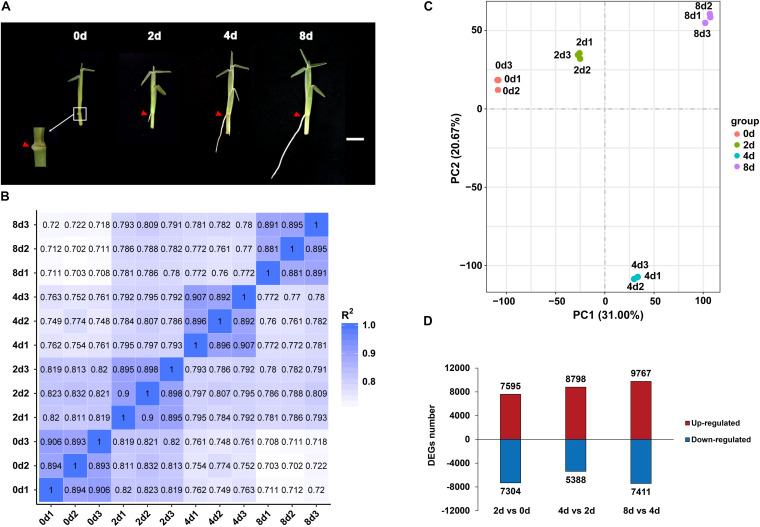
Expression profiles of centipedegrass E039 nodal roots. **(A)** The first nodal roots of E039 were exhibited in 0 day (the root length about 0.1 cm), 2 days (the root length about 0.5 cm), 4 days (the root length about 2.0 cm), and 8 days (the root length about 4.0 cm) after water culture. The roots are indicated by red triangles. *Scale bar* represents 1 cm. **(B)** Pearson’s correlation between 12 samples. **(C)** PCA of 12 transcriptome samples. **(D)** The number of upregulated and downregulated DEGs in 2 days vs. 0 day, 4 days vs. 2 days, and 8 days vs. 4 days.

The rooting parts of centipedegrass are primarily in the nodes of stolons. To determine the node order in the stolon, we defined the node that has three leaves as the first node (Figure 1A). The following nodes were the second node, third node, fourth node, and so on (Figure 1A). To identify the node order for transcriptome sequencing research, we evaluated the rooting ability of nodes 1–16 (from young nodes to old nodes) from E039 (Figure 1B). The rooting rates of nodes were counted after 4, 8, and 12 days of water culture. The results showed that nodes 1–4 had a higher rooting rate, especially the first node, which had the highest rooting rate (Figure 1C). Following the fifth node, the rooting rates decreased significantly (Figure 1C). The rooting rate statistic of lateral branches showed that branch rooting was dominant in nodes 5–16 (Figure 1D). These results indicated that the young nodes of centipedegrass rooted more easily, while the old nodes rooted with greater difficulty. The lateral branches appear in the old nodes and develop into new stolons. Therefore, we chose the first node of centipedegrass E039 for subsequent research. The roots elongated during water culture in 0, 2, 4, and 8 days (Figure 2A).

### Transcriptome Sequencing of Centipedegrass E039 Nodal Roots

Root samples of the first nodes for RNA-seq were collected at 0, 2, 4, and 8 days of water culture. Each sample had three biological repeats. An average of 66.6 million raw reads from the 12 libraries were obtained, and the Q20 (proportion of nucleotides with a quality value greater than 20 in clean reads) percentage range was determined to be 95.04–97.46% (Supplementary Table 3). In total, 199,988 unigenes were revealed by RNA-seq assays. These unigenes had the highest annotation ratio of 72.36% in Nt, and the annotated unigenes were primarily classified in *Sorghum bicolor* and *Zea mays* (Supplementary Figure 1), indicating that centipedegrass had close genetic relationships with sorghum and maize. There were more unigenes (119,448, 59.73%) with lengths exceeding 1,000 bp than unigenes (80,540, 40.27%) with lengths between 200 and 1,000 bp. The mean length of unigene sequences was 1,699 nucleotides (nt), and the N50 was 2,517 nt. The unigene expression levels of replicate samples were estimated from the value of fragments per kilobase per million fragments (FPKM) and highly corrected with Pearson correlation coefficients between 0.88 and 0.91 (Figure 2B). A principal component analysis (PCA) of all the unigenes indicated that 12 samples clustered four groups, and the 4 day time point group was notably distinct from the other time points (Figure 2C).

### Differential Expression During Nodal Root Elongation of Centipedegrass

The DEGs were identified from each comparison with padj < 0.05 and | log2FoldChange| > 1. In total, 33,561 unigenes were differentially expressed among the 2 days vs. 0 day, 4 days vs. 2 days, and 8 days vs. 4 days pairwise comparisons. In the 2 days vs. 0 day comparison, 7,595 unigenes were upregulated, and 7,304 unigenes were downregulated (Figure 2D). The numbers of upregulated DEGs were increased in 4 days vs. 2 days and 8 days vs. 4 days, with 8,798 and 9,767 genes being upregulated, respectively (Figure 2D). The number of downregulated DEGs was decreased at 4 days vs. 2 days (5,388 genes) and was increased at 8 days vs. 4 days (7,411 genes) (Figure 2D).

To explore the molecular mechanism underlying nodal root development, the functional categories of the DEGs were classified through GO analysis. The top 30 enriched GO terms of the 2 days vs. 0 day comparison showed that DEGs were primarily enriched in “oxidation–reduction process,” “regulation of RNA biosynthetic process,” “regulation of transcription, DNA-templated,” “regulation of nucleic acid-templated transcription,” “oxidoreductase activity,” and “response to stress” (number of genes > 1,000) (Supplementary Figure 2A and Supplementary Table 4). The top 30 enriched GO terms of the 4 days vs. 2 days and 8 days vs. 4 days comparisons showed the same results: DEGs were primarily enriched in “oxidation–reduction process” and “oxidoreductase activity” (number of genes > 1,000) (Supplementary Figures 2B,C and Supplementary Table 4).

**FIGURE 3:**
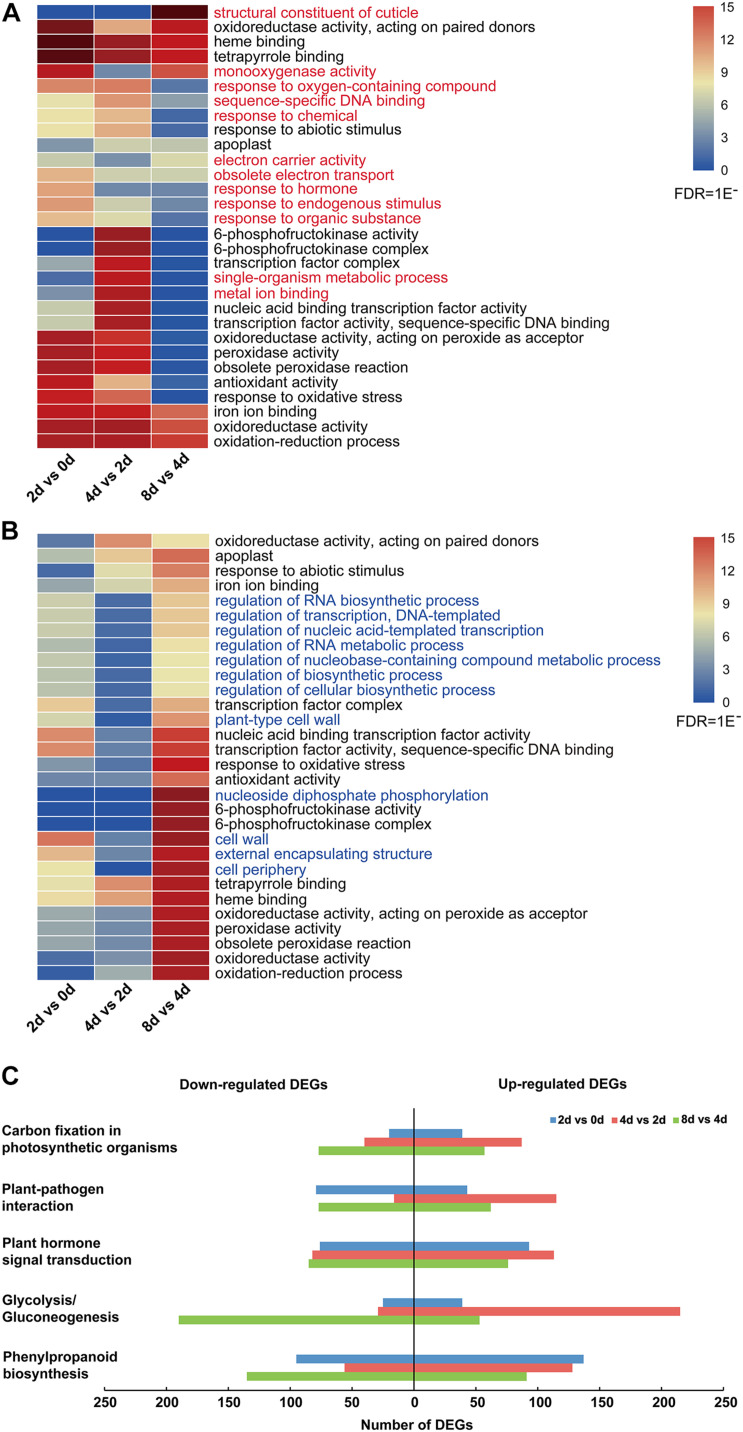
DEG analysis of centipedegrass nodal root development stages. **(A)** Top 30 enriched GO terms of all upregulated DEGs. **(B)** Top 30 enriched GO terms of all downregulated DEGs. **(C)** Number of upregulated and downregulated DEGs enriched in five KEGG pathways.

Moreover, the top 30 enriched GO terms of all upregulated and downregulated DEGs showed that the common enrichments were primarily related to oxidation–reduction processes, including “oxidoreductase activity, acting on paired donors,” “oxidoreductase activity, acting on peroxide as acceptor,” and “peroxidase activity” (Figures 3A,B and Supplementary Table 5). In addition, transcription factor (TF)-related terms were clearly enriched, including “transcription factor complex,” “nucleic acid binding transcription factor activity,” and “transcription factor activity, and sequence-specific DNA binding” (Figures 3A,B and Supplementary Table 5). Moreover, 12 enriched GO terms were specifically enriched in upregulated and downregulated DEGs (Figure 3). Among these genes, those related to the “response to hormone” GO term were especially upregulated in the 2 days vs. 0 day comparison (Figure 3A). For all downregulated genes, the top 30 enriched GO terms were nearly significantly different in the 8 days vs. 4 days comparison (Figure 3B).

To characterize the DEG functions, pathways significantly involved (*P*-value < 0.05) in the response to nodal root development were identified using the KEGG database. For the upregulated and downregulated DEGs, a total of 29 and 19 KEGG pathways were significantly enriched, respectively (Supplementary Table 6). The pathways containing the above five most DEGs were “phenylpropanoid biosynthesis,” “glycolysis/gluconeogenesis,” “plant hormone signal transduction,” “plant–pathogen interaction,” and “carbon fixation in photosynthetic organisms” (Supplementary Table 6 and Figure 3C). In these five KEGG pathways, the number of upregulated DEGs was clearly greater than that of downregulated DEGs in the 4 days vs. 2 days comparison, while the number of upregulated DEGs was clearly less than that of downregulated DEGs in the 8 days vs. 4 days comparison (Figure 3C). Meanwhile, the numbers of upregulated DEGs in “glycolysis/gluconeogenesis,” “plant hormone signal transduction,” “plant–pathogen interaction,” and “carbon fixation in photosynthetic organisms” pathways exhibited an increasing trend from the 2 days vs. 0 day comparison to the 4 days vs. 2 days comparison and exhibited a decreasing trend from the 4 days vs. 2 days comparison to the 8 days vs. 4 days comparison (Figure 3C).

### Identification of DEGs Involved in the Centipedegrass Nodal Root Development

To further screen DEGs, the selection conditions were set as | log2FoldChange| > 2 and padj < 0.05. The results of GO and KEGG analyses indicated that plant hormone signal transduction might play an important role in centipedegrass nodal root development. A total of 201 DEGs were identified in plant hormone signal transduction and selected from all of those comparisons (Supplementary Table 7). Ubiquitin-mediated proteolysis participates in multiple signal transduction pathways of plant hormones, including auxin, gibberellin, ethylene, and jasmonic acid^7^. Therefore, 293 DEGs of E3 ubiquitin-protein ligases were selected from all of those comparisons (Supplementary Table 8). In addition, TF families may also play important roles in centipedegrass nodal root development, and 923 DEGs of TF families were identified from all of those comparisons (Supplementary Table 9). After the trend analysis, the clearly upregulated (profile 4 and profile 5) DEGs and downregulated (profile 0 and profile 1) DEGs were set aside for further analysis (Supplementary Figure 3).

**FIGURE 4:**
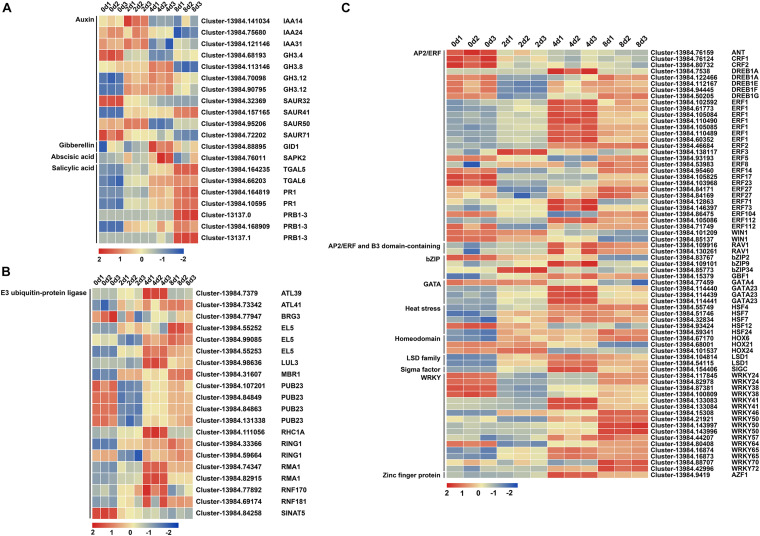
Heatmap of 109 DEGs at four time points after water culture. The 109 DEGs were grouped into **(A)** “plant hormone signal transduction,” **(B)** “E3 ubiquitin-protein ligase,” and **(C)** “transcription factor” categories. The red (upregulated) and blue (downregulated) rectangles are shown with the scale of log2FPKM of each gene.

After reconfirming the selected DEG functions and removing the DEGs with particularly low expression levels (FPKM value < 10 in all samples), 20, 20, and 69 DEGs were reserved in plant hormone signal transduction, E3 ubiquitin-protein ligase, and TF, respectively (Supplementary Table 1). In plant hormone signal transduction, three, four, and five DEGs belonged to *the auxin/INDOLE-3-ACETIC ACID* (*AUX/IAA*), *auxin-responsive GH3 family protein* (*GH3*), and *small auxin-up RNA* (*SAUR*) families in auxin signal transduction, respectively (Supplementary Table 1 and Figure 4A). Among these genes, five DEGs were upregulated, while six DEGs were downregulated (Supplementary Table 1 and Figure 4A). Salicylic acid signal transduction exhibited seven upregulated DEGs, including two *TGA* family members and five *PR1* (*pathogenesis-related protein 1*) family members (Supplementary Table 1). Gibberellin (GA) signal transduction yielded one upregulated gene, *GIBBERELLIN INSENSITIVE DWARF1* (*GID1*), and abscisic acid (ABA) signal transduction resulted in one upregulated gene, *osmotic stress/ABA-activated protein kinase 2* (*SAPK2*), during centipedegrass nodal root development (Supplementary Table 1). In ubiquitin-mediated proteolysis, 20 DEGs of E3 ubiquitin-protein ligase were selected, 12 of which were upregulated and eight of which were downregulated (Supplementary Table 1). However, most of the E3 ubiquitin-protein ligase DEGs exhibited an initial upregulation followed by a decreasing tendency or an initial downregulation followed by an increasing tendency (Figure 4B). Only *Cluster-13984.84258* (*SINAT5*) had a clear decreasing tendency during root development at the four time points (Figure 4B). Meanwhile, 69 DEGs were identified in nine TF families, and the most strongly enriched family was the *APETALA2/ETHYLENE RESPONSE FACTOR* (*AP2/ERF*) domain class transcription factor with 33 DEGs (Supplementary Table 1 and Figure 4C). The second most enriched family was *WRKY* TFs, containing 16 DEGs (Supplementary Table 1 and Figure 4C). The rest of the DEGs belonged to the *basic (region) leucine zipper* (*bZIP*) TF family (four DEGs), *GATA* TF family (four DEGs), *heat stress* TF family (five DEGs), *homeodomain* TF family (three DEGs), *lysine-specific demethylase* (*LSD*) TF family (two DEGs), *sigma factor* TF (one DEG), and *zinc finger proteins* (one DEG) (Supplementary Table 1 and Figure 4C). These results showed that auxin signal transduction, E3 ubiquitin-protein ligases (especially *Cluster-13984.84258* and SINAT5), the *AP2/ERF* TF family, and the *WRKY* TF family may have significant roles in regulating centipedegrass nodal root development.

### Verification of RNA-Seq Data

To confirm the reliability of the RNA-seq data, we selected 12 genes from the 109 DEGs (Supplementary Table 1) and validated them using quantitative real-time PCR (qRT-PCR). These DEGs were significantly upregulated or downregulated during centipedegrass nodal root growth (Supplementary Figure 4). The qRT-PCR results were largely consistent with the RNA-seq data, demonstrating that our sequencing data for centipedegrass nodal roots were reliable.

### Ectopic Expression of EoSINAT5 and Knockout of the Homologous Gene *LOC_Os07g46560* in Rice Affected Root Development

**FIGURE 5:**
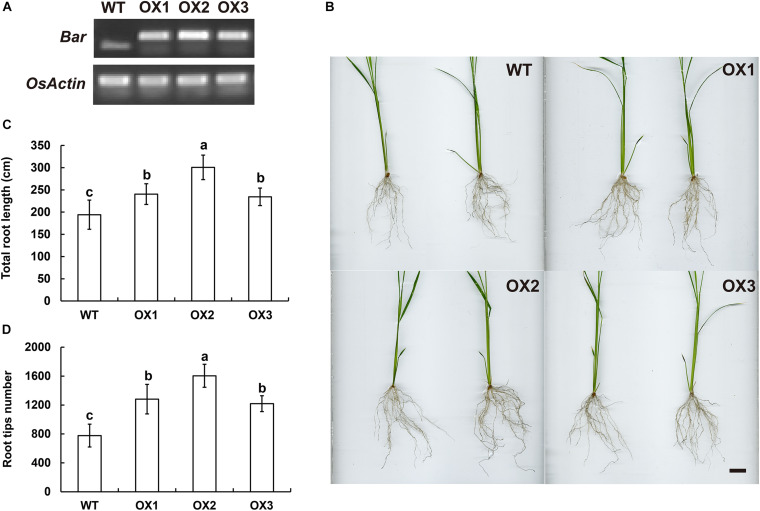
Ectopic expression of *EoSINAT5* in “Nipponbare” rice. **(A)** RT-PCR analyses of T3-overexpressing *EoSINAT5* transgenic rice lines. The reference gene is *OsActin*. The resistance marker gene is *Bar*. **(B)** Root phenotypes of WT and *EoSINAT5-*overexpressing transgenic plants. *Scale bar* represents 2 cm. **(C)** Total root length of WT and *EoSINAT5*-overexpressing transgenic plants. **(D)** Root tip numbers of WT and *EoSINAT5*-overexpressing transgenic plants. Letters above bars indicate significant differences between the respective values (*p* < 0.05).

**FIGURE 6:**
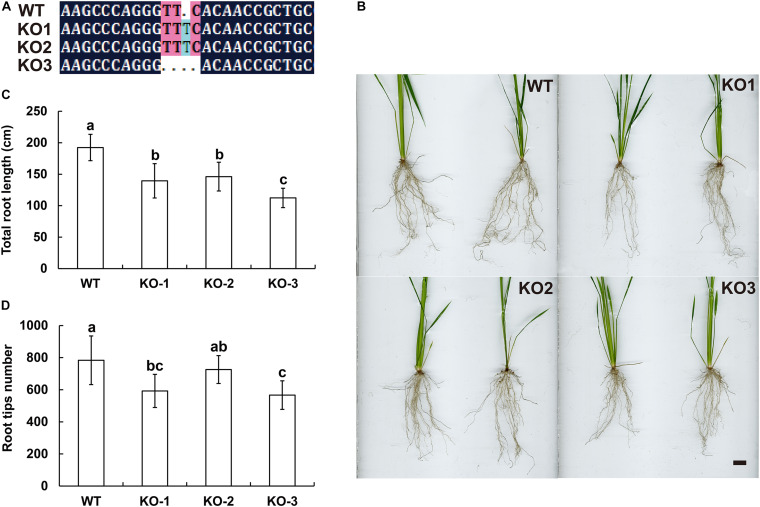
Knocking out *LOC_Os07g46560* (*EoSINAT5* homologous gene) in “Nipponbare” rice. **(A)** Sequencing analyses of *LOC_Os07g46560* knockout homozygous lines. **(B)** Root phenotypes of WT and *LOC_Os07g46560* knockout homozygous lines. *Scale bar* represents 1 cm. **(C)** Total root length of WT and *LOC_Os07g46560* knockout homozygous lines. **(D)** Root tip numbers of WT and *LOC_Os07g46560* knockout homozygous lines. Letters above bars indicate significant differences between the respective values (*p* < 0.05).

The genomic sequences of *SINAT5* between *E. ophiuroides* and *Arabidopsis thaliana* were compared, and two introns were contained in *EoSINAT5* (*Cluster-13984.84258*), while only one intron was observed to be contained in *AtSINAT5* (*AT5G53360*, see text footnote 4) (Supplementary Figure 5A). The amino acid sequence alignment among *A. thaliana*, *E. ophiuroides*, and other Poaceae plants showed that EoSINAT5 has RING-HC and Sina domains, and these two domains were conserved in Poaceae plants (Supplementary Figure 5B). However, compared with *A. thaliana*, the RING-HC and Sina domains of SINAT5s in Poaceae plants had multiple amino acid site differences (Supplementary Figure 5B), indicating that SINAT5s in Poaceae plants might have function differences. During nodal root development in centipedegrass, *EoSINAT5* showed a clearly tendency of expression reduction (Figure 4B). To further examine gene function, *EoSINAT5* driven by the cauliflower mosaic virus (CaMV) 35S promoter was introduced into rice “Nipponbare” by Agrobacterium-mediated genetic transformation. In total, 16 transgenic lines were obtained, and 11 lines exhibited similar phenotypes. Three of 11 transgenic lines were chosen to analyze the root length and root tip number. Compared with wild-type (WT) plants, the transgenic lines had longer roots and more numerous root tips (Figure 5). Furthermore, we knocked out *LOC_Os07g46560* the homologous gene of *EoSINAT5* in rice and obtained five mutant transgenic lines. One base deletion line and two base insertion lines were chosen for further analysis. Compared with WT plants, the mutant transgenic lines had shorter roots and fewer root tips (Figure 6). These results showed that *EoSINAT5* promoted root development and that its homologous gene had a similar function in modern plant rice. In *Arabidopsis*, the E3 ubiquitin-protein ligase SINAT5 ubiquitinates NAC1 to participate in auxin signal transcription and regulate root development (Xie et al., 2002). In our transcriptome data, expression of two *NAC1* unigenes was found upregulated evidently, which shows the contrary expression trend of *EoSINAT5* (Supplementary Table 10).

## Discussion

### Aging of Nodes Limited the Rooting Ability of Centipedegrass

Centipedegrass is an important warm-season grass that reproduces primarily through developed stolons. The node rooting ability of stolons is a significant factor limiting lawn establishment. Previous studies showed that aging is a limiting factor for adventitious rooting competence (Bellini et al., 2014). In *Arabidopsis*, the derooted hypocotyls of young (12-day-old) plants root readily within a week, while adult (26-day-old) plants need a longer time to root and still root poorly (Díaz-Sala et al., 2002). In our research, a similar rooting pattern was observed in centipedegrass. The young nodes of centipedegrass rooted more easily, while the old node’s rooting showed greater difficulty (Figures 1C,D). However, in the actual lawn establishment process, the swards contain numerous old nodes, which greatly affects the speed of centipedegrass grass turf planting.

### Transcriptome Sequencing of Centipedegrass Nodal Roots

Adventitious root development is mainly divided into three phases, including induction, initiation, and expression (Geiss et al., 2018). The transcriptome sequencing samples of first nodal roots at four time points (0, 2, 4, and 8 days) after water culture belong to the expression phase of root emergence. According to the root morphology in different sampling time points (Figure 2A), the 0 and 2 day roots might be in the early emerging phase, when they still lack an elongation zone (Motte et al., 2019), whereas, the roots elongate obviously from 2 to 4 days (Figure 2A), indicating that roots might enter a phase of rapid elongation with the formation of elongation zone. After transcriptome sequencing, the PCA showed that the 4 days group had the highest dispersion degree compared with the other three groups (Figure 2C), and the GO enrichment analysis showed that DEGs related to “cell wall” and “plant-type cell wall” were especially downregulated in the 8 days vs. 4 days comparison (Figure 3B). These results indicate that 4 days might be an important time point relating to the root elongation zone formation.

Previous studies have showed that multiple plant hormones perform coordinated regulation in root development, and auxin stands out as a key instructive signal (Motte et al., 2019). In the root elongation zone, auxin promotes cell expansion by coordinating the activity of SAURs, *Arabidopsis* H+-ATPase (AHAs) and cell wall-modifying proteins (Motte et al., 2019). In our research, GO enrichment and KEGG pathway analyses showed that DEGs enriched significantly in plant hormone signal transduction (Figures 3A,C), indicating that plant hormones might have important effects on centipedegrass nodal root development. In addition, DEGs were clearly enriched in oxidation–reduction process by GO enrichment analysis (Figure 3A). Reactive oxygen species (ROS) signaling affects root development by stiffening the cell wall to inhibit cell expansion, and is not dependent on auxin (Motte et al., 2019). Therefore, we focused on the DEGs in plant hormone signal transduction for the moment.

### Plant Hormone Signal Transduction Involved in Nodal Root Development

In total, 20 DEGs were determined to be associated with plant hormone signal transduction. Among these genes, 11 DEGs belonged to auxin signal transduction, one DEG belonged to gibberellin signal transduction, one DEG belonged to abscisic acid signal transduction, and seven DEGs belonged to salicylic acid signal transduction (Supplementary Table 1 and Figure 4A). These results showed that in addition to auxin signal transduction, salicylic acid signal transduction might also have important effects on root development. Furthermore, 14 DEGs were upregulated, and six DEGs were downregulated, during centipedegrass nodal root development (Supplementary Table 1).

In auxin signal transduction, three *AUX/IAA* family genes (*IAA14*, *IAA24*, and *IAA31*) were identified (Supplementary Table 1). Previous studies in *Arabidopsis* have shown that *iaa14* mutant plants have no lateral root initiation sites and no lateral roots (Vanneste et al., 2005). IAA14 can interact with ARF7 and ARF19 to inhibit the activities of these ARFs (Fukaki et al., 2005). In rice, the expression level of *IAA24* is decreased in the roots of *OsDGK1*-overexpression transgenic plants which exhibit lower lateral root density and thicker seminal roots (Yuan et al., 2019). *IAA31* is known to function in lateral root formation. In rice, overexpression of *OsIAA31* leads to defects in lateral root initiation and lateral root reduction (Nakamura et al., 2006). In our research, *IAA14* was upregulated, while *IAA24* and *IAA31* were downregulated, during root development, indicating that *IAA14* and *IAA24* might have positive effects, while *IAA31* might have negative effects, on centipedegrass nodal root formation.

The *GH3* gene family is generally regulated during plant development steps, such as root and hypocotyl growth. In *Arabidopsis*, overexpression of *GH3.2* (*YDK1*) and *GH3.5* (*WES1*) in transgenic plants disrupts adventitious root development and reduces primary root growth (Takase et al., 2004; Park et al., 2007b). The *GH3.6* gene can affect multiple phenotypes of plants, including shoots, roots, and hypocotyls (Nakazawa et al., 2001). The function of *GH3.9* is to regulate primary root development, and *gh3.9* mutants display elongated roots compared with WT plants (Tatematsu et al., 2008). Several *GH3* genes also exert an effect in balancing auxin homeostasis (Chapman and Estelle, 2009). However, the function of *GH3.4*, *GH3.8*, and *GH3.12* in root growth has not been determined. In our research, one upregulated gene, *GH3.4*, and three downregulated genes (one *GH3.8* and two *GH3.12*) might play important roles in centipedegrass nodal root development.

The *SAUR* gene family is an auxin-responsive gene family that plays an important role in auxin-induced acid growth steps, such as cell elongation and growth adaptation (Stortenbeker and Bemer, 2018). *AtSAUR32* is the first characterized *SAUR* gene in *Arabidopsis*, and the overexpression of *AtSAUR32* in plants reduces hypocotyl elongation and causes defects in apical hook maintenance (Park et al., 2007a). The *SAUR32* gene in centipedegrass was obviously downregulated during nodal root growth, indicating that *EoSAUR32* might inhibit cell elongation in order to reduce root elongation. Plants overexpressing *AtSAUR41* exhibit elongated hypocotyls, increased length of primary roots, and increased number of lateral roots (Kong et al., 2013). In our research, the *EoSAUR41* gene was upregulated during root growth, indicating that it had similar functions to *AtSAUR41* in promoting centipedegrass nodal root development. The *SAUR71* gene belongs to the *SAUR41* subfamily, which has different amino acid sequences in the N-terminus from other *SAUR* families (Kong et al., 2013). However, the expression trend of *EoSAUR71* was contrary to that of *EoSAUR41*, indicating that *EoSAUR71* might have the opposite function as *EoSAUR41*. At present, a *SAUR50-like* gene of sunflower has been reported to be specifically expressed on the eastern side of the stem and is related to the diurnal bending of the apex toward the sun (Atamian et al., 2016). However, to the best of our knowledge, the function of *SAUR50* in root development has not been reported to date. In our research, the *SAUR50* gene was downregulated in root growth, showing that this gene might play important roles in roots.

Salicylic acid (SA) is well known for improving plant resistance by inducing expression of pathogenesis-related proteins; also, it has important roles in plant growth and development (Pasternak et al., 2019). In *Arabidopsis*, adding of low-concentration exogenous SA (below 50 μM) promotes adventitious roots and changes the root apical meristem architecture, while high-concentration exogenous SA (higher than 50 μM) inhibits root development from all growth processes (Pasternak et al., 2019). Furthermore, exogenous SA treatments change the auxin synthesis and transport in plants (Pasternak et al., 2019). In SA signal transduction, functions of the *TGAL5* and *TGAL6* genes have not been fully elucidated. Previous studies in rice showed that TGAL5 interacts with NPR1 (non-expressor of pathogenesis-related genes 1) homologs NH4 and NH5 and is predicted to be involved in plant development (Chern et al., 2014). *PR1/PRB1* genes have been utilized as marker genes for plant defense responses (Mitsuhara et al., 2008). Among 22 *Arabidopsis PR1* genes, only one gene is pathogen-inducible, while most *PR1* genes in rice can be induced by pathogens (Mitsuhara et al., 2008). The *Arabidopsis drought-induced 19* (*Di19*) gene can bind to the *PR1* promoter to enhance drought tolerance, and *PR1*-overexpressing plants have a drought-tolerant phenotype (Liu et al., 2013). In our research, all *PR1*/*PRB1* genes were upregulated, indicating that these genes might be involved in nodal root development in centipedegrass.

Gibberellin is necessary for the development of multiple organs and its deficiency can cause reduced elongation of primary roots by reducing cell elongation and proliferation rate (Rizza et al., 2017). However, GA treatment seems to negatively affect the adventitious root formation and reduce the number of adventitious roots (Bellini et al., 2014; Mauriat et al., 2014). However, synergistic effect of GA and ethylene have been proved in promoting adventitious root formation, and abscisic acid (ABA) can act as a competitor of GA in synergistic effect with ethylene, which leads to negative regulation in adventitious root development in tomato and rice (Bellini et al., 2014). Exogenous ABA has either positive or negative effects on root growth depending on its concentration (Li et al., 2017). *GID1* is the endogenous GA receptor, and the GA–GID1 complex can interact with DELLA proteins, which negatively regulate the GA signaling pathway (Voegele et al., 2011). The GA-GID1-DELLA complex can be recognized by the E3 ubiquitin ligase complex SCF^SLY1/GID2^ and can be degraded through the 26S proteasome (Voegele et al., 2011). Mutation of the *GID1* gene in *Arabidopsis* gives rise to shortened roots and hypocotyls (Griffiths et al., 2006). In centipedegrass, the *GID1* gene was upregulated indicating that this gene might have positive effects during root development. In ABA signal transduction, SAPK2 is a member of sucrose non-fermenting-1-related protein kinase 2 (SnRK2) subclass II (Kim et al., 2012). In rice, the *sapk2* mutant and *SAPK2*-OE plants have similar root lengths as WT plants, while after NaCl treatment, the *sapk2* mutants have decreased root length, and *SAPK2*-overexpression plants clearly exhibit increased root length (Lou et al., 2018). Therefore, the upregulated *SAPK2* gene might have a positive function in centipedegrass nodal root development.

### E3 Ubiquitin-Protein Ligase Involved in Nodal Root Development

The ubiquitin-proteasome system (UPS) mediates proteolysis by ubiquitin and is involved in nearly every aspect of plant biology. Ubiquitin-mediated proteolysis involves a three-step enzymatic cascade between E1, E2, and E3 enzymes, and E3 ubiquitin-protein ligases determine the specific recognition of target proteins (Kelley and Estelle, 2012). The E3 ubiquitin-protein ligases have been classified into four groups (i.e., HECT, RING, U-box, and cullin-RING) (Kelley and Estelle, 2012), and DEGs we identified in E3 ubiquitin-protein ligases mostly belong to the RING-finger type, except *PUB23*, which belongs to the U-box type. However, most of these genes have not been reported to be associated with root development, except *EL5* (*Elicitor 5*) and *SINAT5*. In rice, the inactivated EL5 protein gives rise to a rootless phenotype with cell death in root primordia, and moderately impaired E3 activity of EL5 can form short crown roots (Koiwai et al., 2007). Further research has proven that ubiquitin ligase EL5 maintains root meristem viability by regulating cytokinin-mediated nitrogen effects (Mochizuki et al., 2014). For the *RHC1A* gene in *Arabidopsis*, the overexpression line roots have severe alterations in root meristem architecture, while T-DNA insertion lines exhibit a short root phenotype (Jiang et al., 2010). In *Arabidopsis*, overexpressing of *SINAT5* results in fewer lateral roots, and mutations in the RING motif of *SINAT5* result in more lateral roots (Xie et al., 2002).

### EoSINAT5 Was Involved in Root Development

Transgenic rice phenotypes verified that *EoSINAT5* had a positive effect on root development and that its homologous gene *LOC_Os07g46560* in rice had similar amino acid sequences and functions (Figures 5, 6 and Supplementary Figure 5B). A previous study reported that NAC1 activates the expression of two downstream auxin-responsive genes *DBP* (*DNA-binding protein*) and *AIR3* (*Auxin-induced in root cultures protein 3*) (Xie et al., 2000), and AtSINAT5 protein can ubiquitinate NAC1 to downregulate auxin signal transduction (Xie et al., 2002). In our transcriptome data, two *NAC1* unigenes, one *DBP* unigene and nine *AIR3* unigenes were found (Supplementary Table 10). Contrary to *EoSINAT5*, the expression levels of NAC1 were obviously upregulated and indicating that EoSINAT5 and NAC1 might have interaction relationship. However, the *DBP* unigene had downregulated expression level and *AIR3* unigenes had very low expression levels (Supplementary Table 10), proving that they were not activated by *NAC1* gene. Accordingly, *EoSINAT5* and *AtSINAT5* might have a different regulatory mechanism in root development, and the molecular mechanism governing the function of *EoSINAT5* requires further study.

## Conclusion

In our research, aging of nodes limited the rooting ability of centipedegrass. The transcriptome sequencing of nodal roots after 0, 2, 4, and 8 days of water culture revealed that 4 days might be an important time point relating to the root elongation. GO enrichment and KEGG pathway analyses of DEGs indicated that plant hormone signal transduction and transcription factors might play important roles in centipedegrass nodal root growth. E3 ubiquitin-protein ligases might be involved in the plant hormone signal transduction with transcription factors to regulate root development. Transgenic results showed that differentially expressed E3 ubiquitin-protein ligase *EoSINAT5* and its homologous gene (*LOC_Os07g46560*) in rice have a similar function in promoting the root growth.

## Data Availability Statement

The datasets presented in this study can be found in online repositories. The names of the repository/repositories and accession number(s) can be found below: https://www.ncbi.nlm.nih.gov/, PRJNA687624.

## Author Contributions

RW, JW, HZ, and HG performed the evaluation experiments. JW, RW, and Jjl performed the transcriptomic analyses and verification experiments. JW designed the experiment. JZ, HW, and Jxl supervised the project. JW and RW participated in writing the manuscript. All authors contributed to the article and approved the submitted version.

## Conflict of Interest

The authors declare that the research was conducted in the absence of any commercial or financial relationships that could be construed as a potential conflict of interest.

## Footnotes

1 http://www.novogene.com/

2 http://www.genome.jp/kegg/

3 http://www.omicshare.com/tools/Home/Soft

4 http://www.arabidopsis.org/

5 http://gsds.cbi.pku.edu.cn/

6 https://blast.ncbi.nlm.nih.gov/Blast.cgi

7 https://www.kegg.jp/kegg-bin/show_pathway?ko04075

## Supplementary Material

Supplementary Figure 1 Annotation of all unigenes in centipedegrass nodal root development. **(A)** The number and ratio of unigenes annotated in seven databases. **(B)** Species classification of unigene annotation in the NR database.

Supplementary Figure 2 Top 30 enriched GO terms of the 2 days vs. 0 day comparison, 4 days vs. 2 days comparison, and 8 days vs. 4 days comparison.

Supplementary Figure 3 Trend analysis of DEGs in “Plant hormone signal transduction,” “E3 ubiquitin-protein ligase,” and “Transcription factor.” The color modules are significantly enriched modules with *p* < 0.05. The same color indicates a similar trend.

Supplementary Figure 4 qRT-PCR validation of 12 genes randomly selected from the 109 DEGs in Supplementary Table 1. Values are presented as the mean ± SE. The column diagrams represent the relative expression levels of genes. The line charts represent the FPKM values of genes.

Supplementary Figure 5 Gene structure and protein alignment of *EoSINAT5*. **(A)** Structural analysis of the *EoSINAT5* gene in *Arabidopsis* and centipedegrass. Black boxes represent exons, and black lines represent introns. **(B)** Protein alignment of SINAT5s from *Sorghum bicolor* (SbSINAT5, XP_002461156.1), *Setaria italica* (SiSINAT5, XP_004958578.1), *Dichanthelium oligosanthes* (DoSINAT5, OEL30966.1), *Oryza sativa japonica group* (OsSINAT5, XP_015644782.1), *Oryza brachyantha* (ObSINAT5, XP_006658066.1), *Brachypodium distachyon* (BdSINAT5, XP_003557906.1), *Triticum urartu* (TuSINAT5, EMS52645.1), *Aegilops tauschii* subsp. *Tauschii* (AsSINAT5, XP_020178176.1), *Arabidopsis thaliana* (AtSINAT5, AT5G53360), and *Eremochloa ophiuroides* (Munro) Hack. (EoSINAT5, Cluster-13984.84258). Green underlining indicates a conserved RING-HC region of SINAT5 proteins. Red underling indicates a conserved Sina region of SINAT5 proteins. Black, red, and blue represent 100, 75, and 50% identity, respectively. Red asterisks represent different amino acid sites between *A. thaliana* and Poaceae plants.

Supplementary Table 1 Selected DEGs of centipedegrass root development.

Supplementary Table 2 Primer sequences.

Supplementary Table 3 Summary of transcriptome sequencing for centipedegrass E039 roots.

Supplementary Table 4 Top 30 enriched GO terms of DEGs in three comparisons.

Supplementary Table 5 Top 30 GO enrichments of the upregulated and downregulated DEGs.

Supplementary Table 6 Pathway classification of the upregulated and downregulated DEGs.

Supplementary Table 7 DEGs involved in plant hormone signal transduction.

Supplementary Table 8 DEGs of E3 ubiquitin-protein ligases.

Supplementary Table 9 DEGs of TFs.

Supplementary Table 10 FPKM values of unigenes NAC1, DBP, and AIR3.
