# Supplementary material for: Use of Transcriptomic Analyses to Elucidate the Mechanism Governing Nodal Root Development in Eremochloa ophiuroides (Munro) Hack
Source: Front Plant Sci. 2021 Apr 23;12:659830. doi: 10.3389/fpls.2021.659830 (PMC8102984; doi:10.3389/fpls.2021.659830)
Supplement: Supplementary Figure 1 — Annotation of all unigenes in centipedegrass nodal root development. (A) The number and ratio of unigenes annotated in seven databases. (B) Species classification of unigene annotation in the NR database. [file Data_Sheet_1.docx]

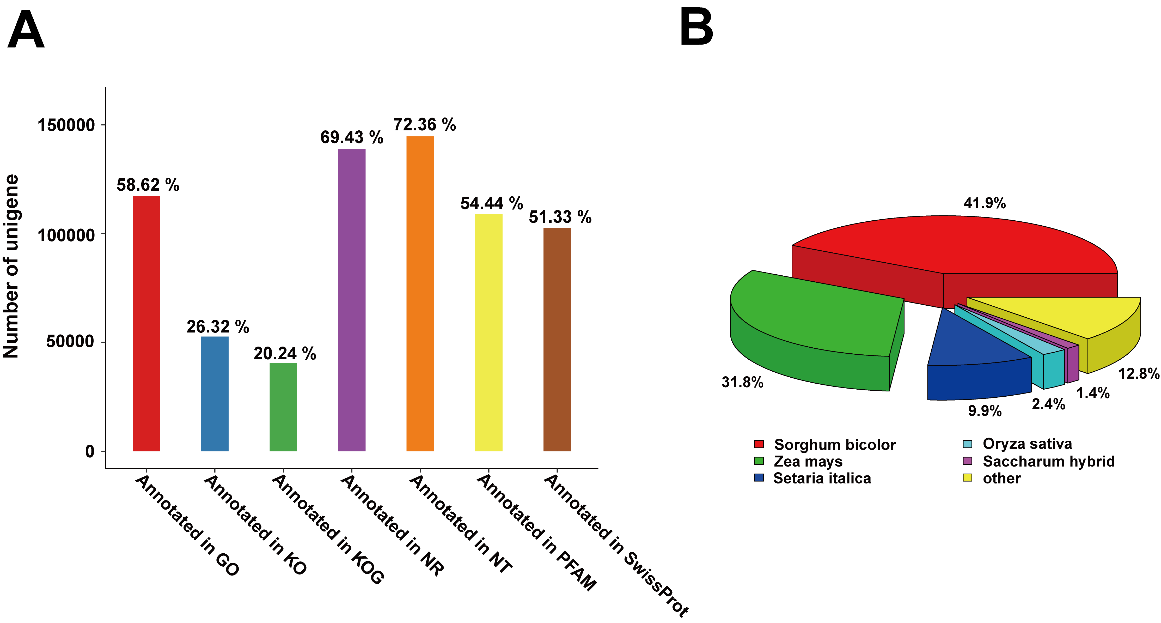


**Figure S1. Annotation of all unigenes in centipedegrass nodal root development. (A)** The number and ratio of unigenes annotated in seven databases. **(B)** Species classification of unigene annotation in the NR database.


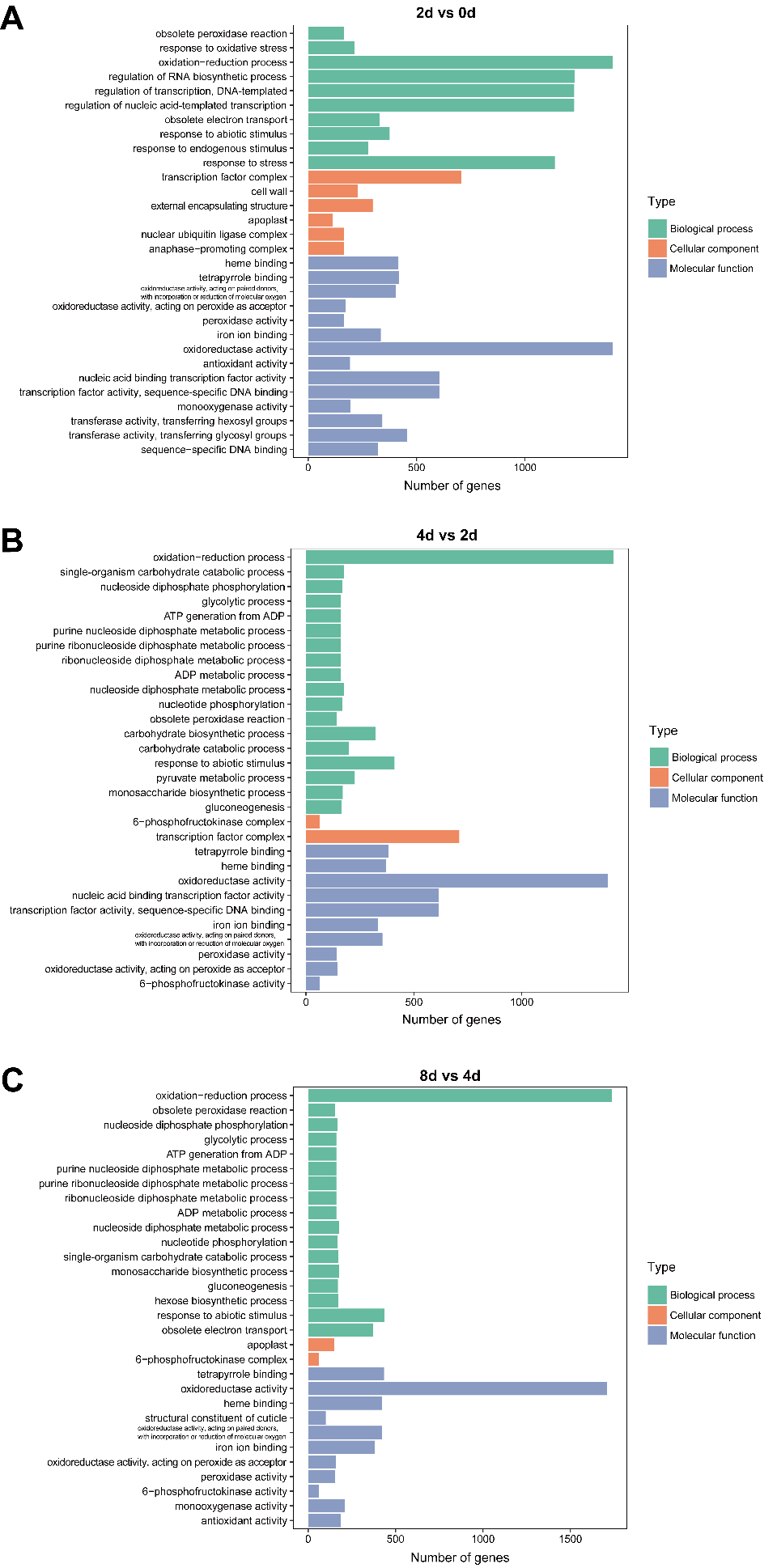


**Figure. S2 Top 30 enriched GO terms of the 2 d vs 0 d comparison, 4 d vs 2 d comparison and 8 d vs 4 d comparison.**


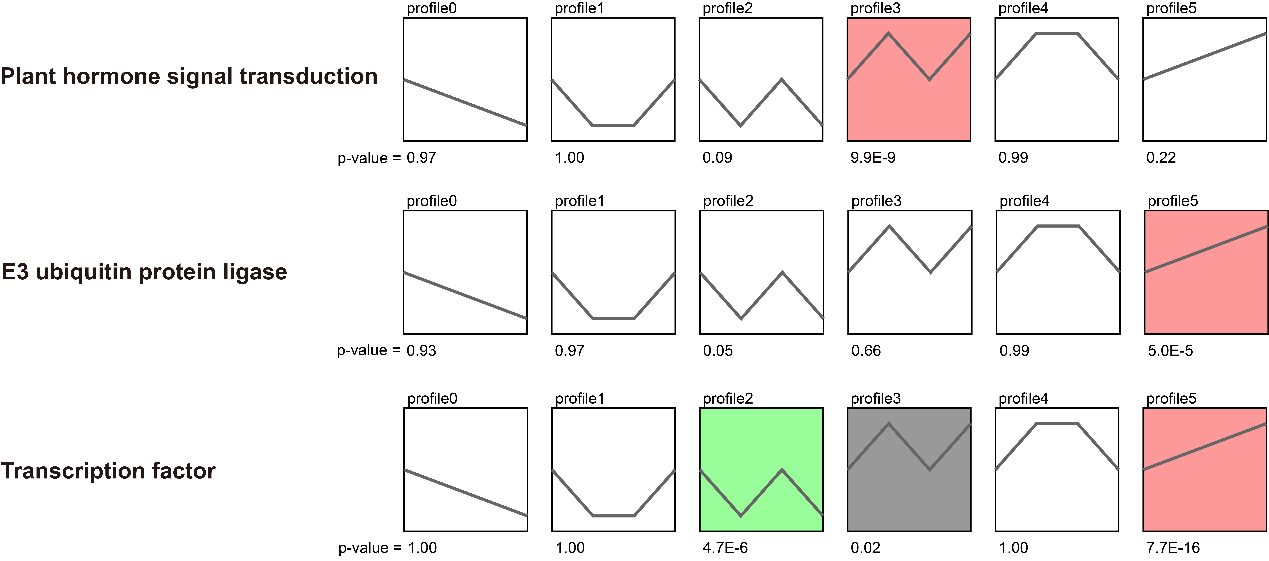


**Figure S3. Trend analysis of DEGs in “Plant hormone signal transduction”, “E3 ubiquitin-protein ligase” and “Transcription factor”. The color modules are significantly enriched modules with p < 0.05. The same color indicates the similar trend.**


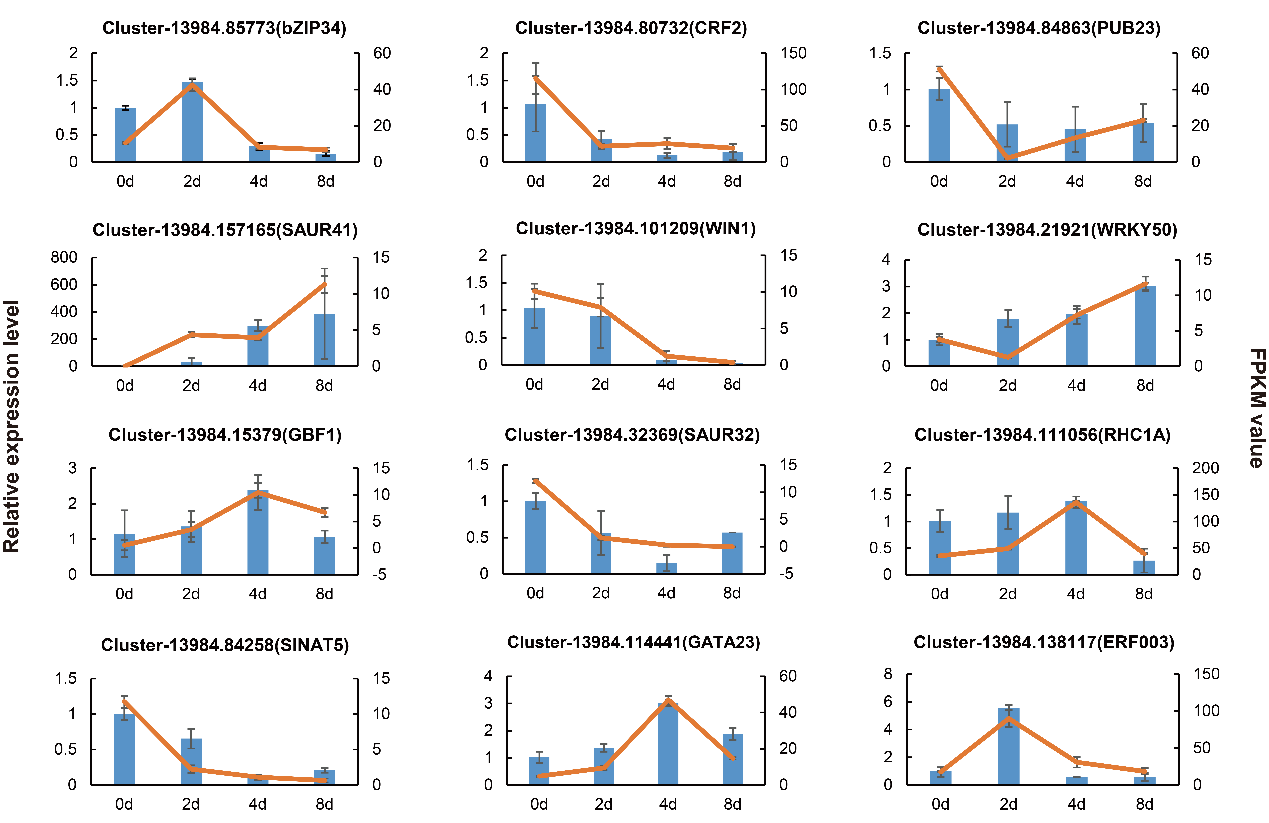


**Figure S4.** **qRT-PCR validation of 12 genes randomly selected from the 109 DEGs in Supplementary Table S1. Values are presented as the mean ± SE. The column diagrams represent the relative expression levels of genes. The line charts represent the FPKM values of genes.**


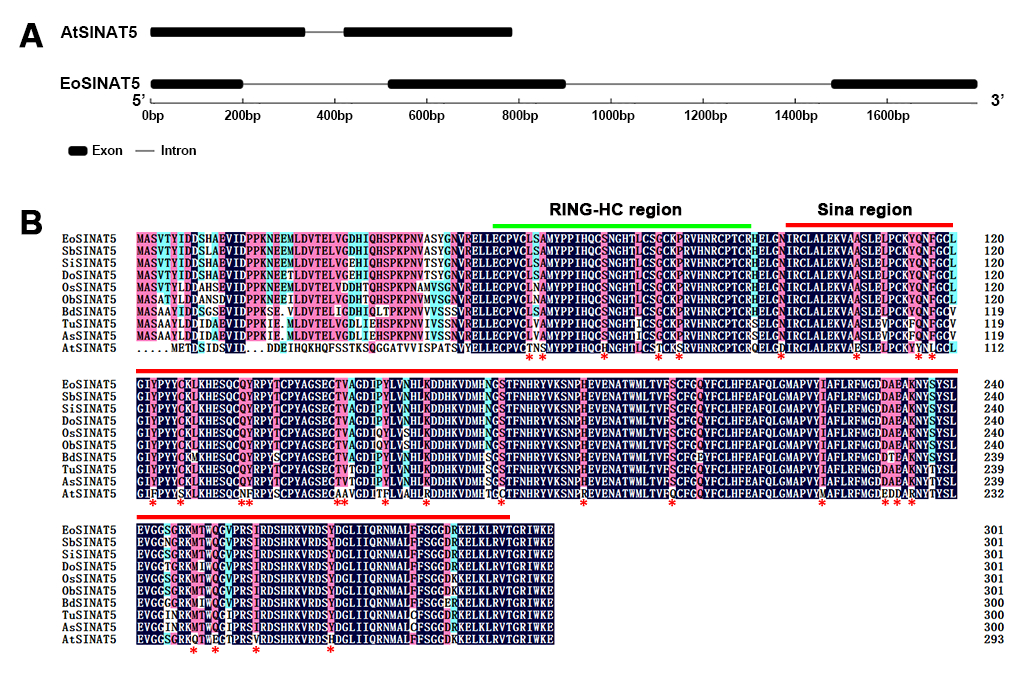


**Figure S5. Gene structure and protein alignment of *EoSINAT5*. (A)** Structural analysis of the *EoSINAT5* gene in *Arabidopsis* and centipedegrass. Black boxes represent exons, and black lines represent introns. **(B)** Protein alignment of SINAT5s from *Sorghum bicolor* (SbSINAT5, XP_002461156.1), *Setaria italica* (SiSINAT5, XP_004958578.1), *Dichanthelium oligosanthes* (DoSINAT5, OEL30966.1), *Oryza sativa japonica group* (OsSINAT5, XP_015644782.1), *Oryza brachyantha* (ObSINAT5, XP_006658066.1), *Brachypodium distachyon* (BdSINAT5, XP_003557906.1), *Triticum urartu* (TuSINAT5, EMS52645.1), *Aegilops tauschii subsp. Tauschii* (AsSINAT5, XP_020178176.1), *Arabidopsis thaliana* (AtSINAT5, AT5G53360) and *Eremochloa ophiuroides* (Munro) Hack. (EoSINAT5, Cluster-13984.84258). Green underlining indicates a conserved RING-HC region of SINAT5 proteins. Red underling indicates a conserved Sina region of SINAT5 proteins. Black, red and blue represent 100%, 75% and 50% identity, respectively. Red asterisks represent different amino acid sites between *A. thaliana* and Poaceae plants.
